# A questionnaire-based survey in Spain provides relevant information to improve the control of ovine coccidiosis

**DOI:** 10.3389/fvets.2023.1326431

**Published:** 2023-12-06

**Authors:** Roberto Sánchez-Sánchez, Jorge Gutiérrez, José Luis Blasco-Castello, María Marcos-Santamaría, Santiago Cano-Alsua, Laura Elvira, Ignacio Ferre, Luis Miguel Ortega-Mora

**Affiliations:** ^1^SALUVET, Animal Health Department, Faculty of Veterinary Sciences, Complutense University of Madrid, Madrid, Spain; ^2^MSD Animal Health, Polígono Industrial El Montalvo, C/Zeppelin, Salamanca, Spain; ^3^Computing Services, Research Support Center, Complutense University of Madrid, Madrid, Spain

**Keywords:** coccidiosis, *Eimeria*, sheep, questionnaire, survey, control, diagnosis, Spain

## Abstract

Ovine coccidiosis is a widespread intestinal parasitic disease caused by *Eimeria* spp. Lambs are infected by the ingestion of sporulated oocysts, experiencing diarrhea and low growth rates. Control should be based on measures to reduce infection pressure and stress on the animals as well as on appropriate diagnosis and strategic treatment. To obtain information on how control measures are implemented in the ovine sector in Spain, a questionnaire-based survey was completed in 2022 by 154 veterinarians and 173 farmers working in this sector. Coccidiosis was highlighted as a relevant disease by 34% of the respondents. The period of greatest risk seemed to differ between production systems, being mainly early after weaning (7–15 days after weaning) in meat flocks and feedlots and later (1–2 months after weaning) in dairy flocks. The absence of cleaning and disinfection measures was identified as a risk factor by 51% of the veterinarians, with 22% mentioning overcrowding of animals and 22% indicating that coccidiosis has more incidence in flocks with large number of animals. The use of laboratory diagnosis methods (fecal oocyst count) was unusual in 70 and 84% of the veterinarians and farmers, respectively. Regarding control, dairy flocks usually housed a larger number of animals under intensive conditions, and they implemented more frequently control measures for coccidiosis than meat flocks. Anticoccidial drugs were used in 79% of the flocks, and in 74–82% of them, they were applied based on clinical criteria. Comparing protocols for anticoccidial treatment among different production systems, in meat flocks, anticoccidial drugs were applied more frequently when clinical signs were observed, and coccidiostats were used for less than 28 days compared to dairy flocks. These results highlight the need for improvement in the use of anticoccidial treatments adjusted to the new regulatory framework in the EU, which in turn will rationalize the use of antimicrobial compounds and may help to mitigate the impact of coccidiosis in flocks.

## 1 Introduction

Ovine coccidiosis is a protozoan intestinal infection caused by coccidia parasites of the genus *Eimeria* (phylum Alveolata, subphylum Apicomplexa, class Coccidea, order Eimeriida, and family Eimeriidae) (1). Ovine coccidiosis is present worldwide (2), and the cumulative incidence in the period around weaning could reach 64–100% (3–6). Coccidiosis is mainly associated with diarrhea, dehydration, reduced voluntary feed intake and other clinical signs that reduce productivity (such as lower average daily weight gain) and may even cause death (7), resulting in very substantial economic losses (8).

Twelve intestinal and one abomasal highly host specific *Eimeria* species have been reported in sheep (7). The life cycle of *Eimeria* species is monoxenous and comprises 3 phases: the first 2 phases are internal and occur in the intestine (asexual and sexual replication), and its development takes approximately 2 weeks (with variations between species), followed by environmental sporogony (sporulation) (7). *Eimeria* species are transmitted by the fecal-oral route, and the sporulated oocyst is the infective stage. The intracellular replication of *Eimeria* sporozoites during asexual stages (merogony) and the subsequent sexual stage (gamogony) damages intestinal cells, although the clinical outcome depends on the three sides of the epidemiological triangle: the host (age, immune status and concurrent infections), the environment (facilities and management practices), and the parasite (different pathogenicity of *Eimeria* spp.). *Eimeria ovinoidalis* and *Eimeria crandallis* affect the distal half of the intestines and are considered major pathogens causing hemorrhagic diarrhea (less often for *E. crandallis*), quickly reaching an almost 100% cumulative incidence in 8 weeks-old lambs (4, 9, 10). On the other hand, most infections with less pathogenic species, such as *Eimeria bakuensis* and *Eimeria ahsata*, remain subclinical unless very high infection doses are ingested. In any case, the presence of several *Eimeria* species (mixed infections) is the most common situation (11, 12). The main risk factors associated with coccidiosis are the age of the animals (mainly between 3 and 8 weeks old), the presence of garbage and muddy zones, overcrowding and the use of pens to house different age groups (regrouping of animals) (6, 13, 14).

Effective control of sheep coccidiosis should be focused on reducing environmental contamination rather than avoiding contact with the parasite (as low-dose infections are not linked with disease and allow hosts to develop a protective immune response) and management practices to reduce stress in the animals (weaning and regrouping of animals) (7). Regarding treatment, in the European Union, only 3 compounds are licensed for the control of ovine coccidiosis, which are decoquinate, toltrazuril and diclazuril (15). Decoquinate is a coccidiostat that has been administered traditionally as a long-term feed additive (16). On the other hand, triazines such as toltrazuril and diclazuril are anticoccidials commercialized as oral suspensions, and their efficacy in reducing fecal excretion and clinical coccidiosis has been demonstrated under natural and experimental conditions (17–19). A very important point to take into consideration in the use of drugs to control coccidiosis is that Regulation (EU) 2019/6 banned the prophylactic use of antimicrobials, recommending the metaphylactic use of anticoccidials after a recent diagnosis of the infection for a short time and in a strategic point in the life cycle of the *Eimeria* species.

Questionnaire-based surveys have been useful in comparatively studying different management systems (20), identifying risk factors associated with lamb mortality (21) or investigating farmers’ perceptions of parasitic diseases (22, 23), including infection by *Eimeria* spp. (24). Coccidiosis is one of the main diseases affecting sheep flocks worldwide, including Mediterranean countries (25); however, there is no information on the approach of veterinarians or farmers to this disease. Therefore, the aim of this study was to conduct a detailed questionnaire-based survey among veterinarians and farmers working in the ovine sector in Spain, as a representative country in the Mediterranean area, to obtain knowledge of *Eimeria* spp. infection dynamics and to identify possible deficiencies in the diagnosis and control of coccidiosis.

## 2 Materials and methods

### 2.1 Design of the survey

Veterinarians were selected by convenience sampling from MSD Animal Health database of Spanish veterinarians of the ovine sector. Farmers were selected by convenience from the clients of the veterinarians in the database. Veterinarians and farmers were contacted by e-mail in 2022 to fill out a questionnaire using the online tool “GetFeedback”.^1^ The questionnaire included different questions about ovine coccidiosis, such as clinical signs, risk factors, disease chronology, diagnosis, and control measures. Farmers were also asked about the characteristics of their flocks, and both veterinarians and farmers were asked about the geographical area in which they worked, the main production of their flocks and the main ovine infectious diseases affecting them. The 18 questions for veterinarians and the 17 questions for farmers and their possible answers are detailed in Table 1. Fifteen of these questions were common to both veterinarians and farmers. Two hundred twenty-eight questionnaires from farmers and 190 questionnaires from veterinarians were completed. All the questionnaires were compiled in Microsoft Excel 2019 (Microsoft Corp., Redmond, WA, United States) for data analysis. Medical research was not carried out; therefore, ethics approval was not needed. Anonymity was maintained in the study; therefore, written informed consent was not needed.

**Table 1:** Questions for veterinarians and farmers on different aspects of ovine coccidiosis.

Category	Questions	Answers
Province	In which Spanish province do you mainly work?^a^	The 52 Spanish provinces
Type of production	In what type of production do you more often work?^a^	Meat flocks Dairy flocks Feedlots
Description of the flocks	Number of animals in the flocks^b^	Meat and dairy flocks: (a) Less than 500 animals (b) 500–1,000 animals (c) 1,000–1,500 animals (d) More than 1,500 animals Feedlots: (a) Less than 2000 lambs (b) 2001–4,000 lambs (c) 4,001–8,000 lambs (d) More than 8,000 lambs
	Replacement rate per year^b^ (for meat and dairy flocks)	(a) Less than 15% (b) 15–25% (c) 25–30% (d) More than 30%
	Replacement lots per year^b^ (for meat and dairy flocks)	(a) 1 lot (b) 2 lots (c) 3 lots (d) 4 lots or more
Main diseases	Select the two diseases affecting sheep flocks that you are most concerned about^a^^,*^	Neonatal diarrhea Ovine respiratory complex Coccidiosis Abortion Others
Clinical signs, timing and risk factors for ovine coccidiosis	What are the two main signs by which you suspect coccidiosis?^a^^,*^	(a) Diarrhea (b) Delay of growth (c) Poor body condition (d) Mortality (e) Immunosuppression
	In which kinds of flocks have you more frequently observed coccidiosis?^c^^,*^	Flocks with large number of animals Flocks with absence of cleaning and disinfection measures for paddocks In lots with problems of overcrowding and heterogeneity Flocks with presence of other diseases Others
	In which period have you observed a greater incidence of coccidiosis?^a^	Before weaning (around one month old) (b) 7–15 days after weaning (c) 1–2 months after weaning (d) At more than 3 months of age (e) There is no specific time point
Diagnosis of ovine coccidiosis	Do you perform coprological studies to monitor coccidiosis?^a^	Not routinely (b) Sporadically (c) Once or twice a year (d) In each lambing season/lot
	Do you order species identification?^c^	No, never (b) Only in the initial diagnosis (c) Yes, routinely
	At what oocyst count do you decide to treat for coccidial infection?^c^	Up to 500 oocysts per gram of feces (opg) (b) 501–1,000 opg (c) 1,001–5,000 opg (d) >5,000 opg
Control of ovine coccidiosis	Which have been your criteria for diagnosing and treating coccidiosis?^a^	Compatible clinical signs without coprological studies Compatible clinical signs and presence of oocysts in feces Presence of oocysts in feces Systemically for all lots without coprological studies
	14) Which treatment/s do you use for coccidiosis?^a^	Oral anticoccidials (b) Medicated feed (c) Combined treatment (medicated feed and oral anticoccidials) (d) I do not usually treat, I only implement management measures (e) I have not needed to apply any treatment (f) Others
	15) When do you apply oral anticoccidials?^a^	One week before weaning At the time of weaning 15 days after weaning When compatible clinical signs appear
	16) How many doses of oral anticoccidials do you apply?^a^	(a) Single dose (b) Two doses (c) Some lots with more than two doses
	17) Who administers the oral anticoccidials?^a^	(a) Farmer (b) Veterinarian (c) Sometimes the veterinarian and sometimes the farmer
	18) How do you calculate the dose of oral anticoccidials?^a^	(a) I weigh several animals to establish an average weight (b) I weigh the heaviest animal (c) I estimate an average weight according to the age of the animals (d) By visual estimation of the weight
	19) When do you start administering medicated feed?^a^	(a) One week before weaning (b) At the time of weaning (c) 15 days after weaning (d) When compatible clinical signs appear
	20) How long do you apply the medicated feed?^a^	(a) Less than 28 days (b) Between 28 and 30 days (c) Between 31 and 45 days (d) Until the lot of feed runs out
	21) Which management measures do you apply?^a^^,*^	(a) Cleaning and disinfection of the paddocks (b) Measures to minimize stress (animal density, weaning, etc.) (c) Moving animals to a new paddock after administering treatment (d) I do not implement management measures
	22) Which disinfectant do you use?^a^^,*^	(a) Peroxides (b) Quaternary ammoniums (c) Others

*Multiresponse question for which a maximum of 2 answers could be selected simultaneously.

a Question asked to both veterinarians and farmers.

b Question asked only to farmers.

c Question asked only to veterinarians.

### 2.2 Criteria for questionnaire inclusion and data analysis

Questionnaires with at least one answered question in the diagnosis section and one answered question in the control section were included in this study. The answers to multi-response questions were grouped into 4–6 categories for statistical analysis (Supplementary File 1). Pearson chi-square tests were performed to study the association between the answers to the different questions asked to veterinarians and asked to farmers and to compare the answers to the common questions between farmers and veterinarians. Statistical significance for all analyses was established at *p* < 0.05. All statistical analyses were performed using GraphPad Prism 8.0.1 software (San Diego, CA, United States). Statistical analysis was not conducted for the questionnaires from feedlots due to the low number of questionnaires available.

## 3 Results

**Figure 1:**
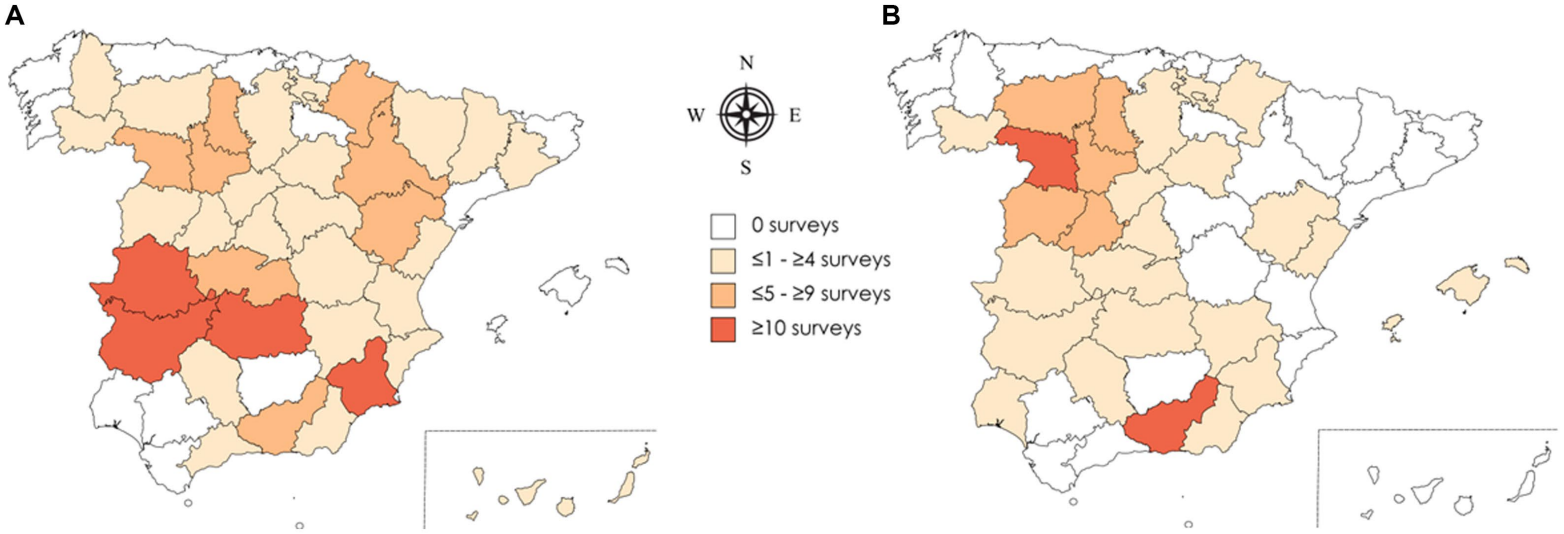
Distribution of the selected questionnaires of the veterinarians (*n* = 154) **(A)** and farmers (*n* = 173) **(B)** by Spanish province. The map of Spain was created by the MapChart online tool (https://mapchart.net/world.html).

One hundred fifty-four questionnaires completed by veterinarians met the inclusion criteria; 76 (49.4%) were from veterinarians mainly working with meat flocks, 65 (42.2%) from veterinarians working mainly with dairy flocks and 13 (8.4%) from professionals working mainly in feedlots. One hundred seventy-three questionnaires completed by farmers met the inclusion criteria: 67 (38.7%) with meat flocks, 102 (59%) with dairy flocks and 4 (2.3%) with feedlots. Questions 1 to 10 and 13 to 21 were answered by 93.5–100% of the respondents, and questions 11, 12, and 22 were answered by 62.7–85.8% of the respondents. The veterinarians and farmers who completed the questionnaires were from 69.2% (36/52) and 51.9% (27/52) of Spanish provinces, respectively (Figure 1).

The comparison of each question with all other questions posed to veterinarians and with all other questions posed to farmers are shown in Supplementary Files 2, 3, respectively. In addition, each shared question between veterinarians and farmers was compared between them (Supplementary File 4). The main results from the questionnaires are shown below.

### 3.1 Description of the flocks

The dairy flocks considered in this study had a larger number of sheep compared to the meat flocks (Table 2). Dairy flocks more frequently included 1,000–1,500 sheep (18.6%, 19/102) compared to meat flocks (6%, 4/67) (*p* < 0.05), which more frequently had less than 500 sheep (38.8%, 26/67) or between 500–1,000 sheep (41.8%, 28/67). In contrast, feedlots were much larger and usually housed more than 8,000 lambs.

**Table 2:** Number of animals in dairy and meat flocks from Spanish farmers surveyed according to the production system.

Category	Dairy flocks	Meat flocks
Less than 500 animals	27.5% (28/102)	38.8% (26/67)
500–1,000 animals	34.3% (35/102)	41.8% (28/67)
1,000–1,500 animals	18.6% (19/102)^a^	6% (4/67)^b^
More than 1,500 animals	19.6% (20/102)	13.4% (9/67)

Different letters (a, b) indicate statistically significant differences between them (*p* < 0.05).

Dairy flocks had a higher replacement rate and replacement lots per year than meat flocks. Concerning the replacement rate, meat flocks more frequently had less than 15% replacement rate per year (31.3%, 21/67) compared to dairy flocks (10.8%, 11/102), which often had a more than 15% replacement rate per year (89.2%, 91/102) (*p* < 0.0001). Concerning replacement lots, meat flocks more frequently had only one lot per year (40.3%, 27/67) compared to dairy flocks (19.6%, 20/102) (*p* < 0.01), which usually had two (28.4%, 29/102), three (16.7%, 17/102) or four or more lots per year (35.3%, 36/102). Comparing the replacement rate and the number of replacement lots per year, the flocks with a lower than 15% replacement rate usually had only one replacement lot (53.1%, 17/32) compared to those with a greater than 15% replacement rate (21.8%, 30/137). On the other hand, flocks with replacement rates greater than 30% usually had 4 replacement lots or more (53.8%, 14/26) (*p* < 0.001).

### 3.2 Main diseases affecting sheep flocks

The main diseases reported in this survey are shown in Table 3. The main diseases reported by a substantially greater percentage of veterinarians (37.6%, 53/141) were neonatal diarrhea and ovine respiratory complex (ORC), compared to farmers (18.9%, 32/169) (*p* < 0.05). On the other hand, the main diseases reported by a substantial percentage of farmers (36.7%, 62/169) were those classified as “others,” compared to veterinarians (7.1%, 10/141) (*p* < 0.0001). Among those responses classified as “others” for farmers, 30.6% (19/62) and 19.4% (12/62) were “only neonatal diarrhea” or “only ORC,” respectively. Coccidiosis was indicated as a relevant disease by 34.8% (49/141) and 33.1% (56/169) of the veterinarians and farmers, respectively.

**Table 3:** Main diseases reported by veterinarians and farmers that affect dairy and meat flocks.

Category	Dairy flock veterinarians	Meat flock veterinarians	Dairy farmers	Meat farmers
ND and ORC	36.9% (24/65)	38.2% (29/76)	24.5% (25/102)	10.4% (7/67)
ND and C or ND and A	36.9% (24/65)^b^	22.4% (17/76)^a^	27.5% (28/102)	26.9% (18/67)
ORC and C or ORC and A	15.4% (10/65)^a^	35.5% (27/76)^b^	23.5% (24/102)	25.4% (17/67)
Others	10.8% (7/65)	3.9% (3/76)	24.5% (25/102)	37.3% (25/67)

ND, neonatal diarrhea; ORC, ovine respiratory complex; C, coccidiosis; A, abortion. Different letters (a, b) indicate statistically significant differences between them (*p* < 0.01).

Regarding production systems, a high percentage of veterinarians (35.5%, 27/76) working mainly with meat flocks were worried about ORC (of them, 70.4%, 19/27, were worried about ORC and coccidiosis and 29.6%, 8/27 about ORC and abortions), while those working with dairy flocks were mainly worried about neonatal diarrhea (36.9%, 24/65; of them, 16/24, 66.6%, were worried about neonatal diarrhea and abortions and 8/24, 33.4%, about neonatal diarrhea and coccidiosis) (*p* < 0.01). In contrast, there were no differences between dairy and meat farmers in their considerations for relevant diseases. For feedlots, 84.6% (11/13) of veterinarians and 100% (4/4) of farmers indicated ORC or ORC and others as the main diseases.

### 3.3 Timing, clinical signs, and risk factors for ovine coccidiosis

**Figure 2:**
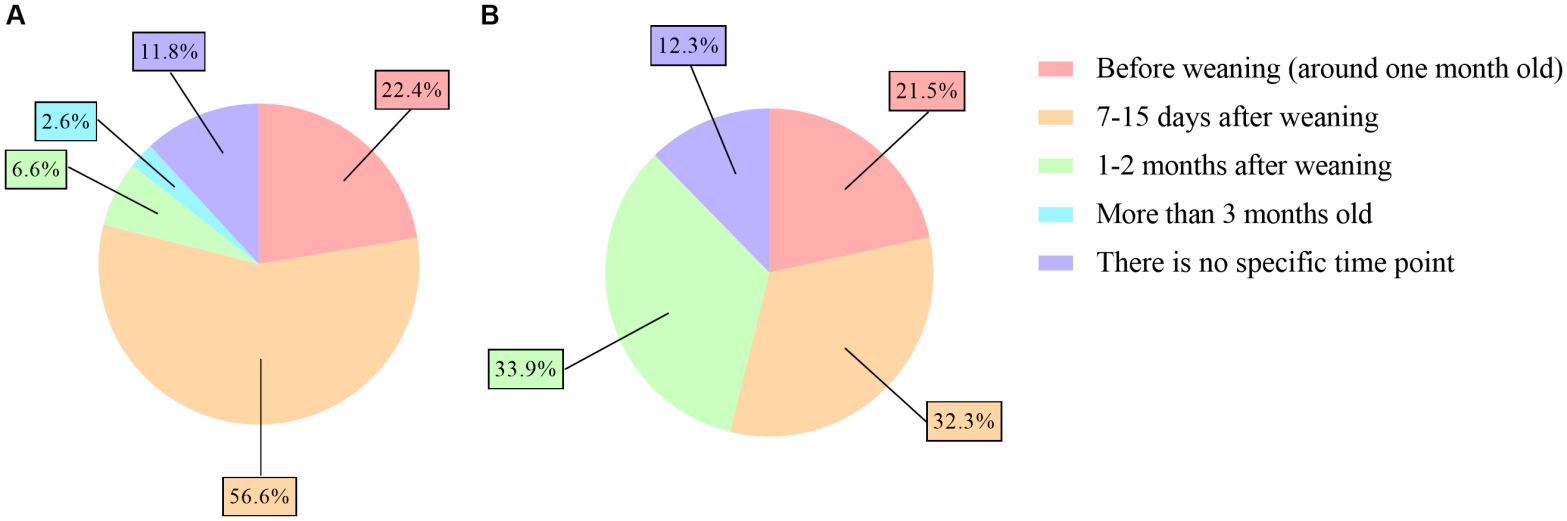
Pie charts of the risk periods of ovine coccidiosis reported by veterinarians in Spanish meat **(A)** and dairy flocks **(B)**.

A significantly higher percentage of veterinarians from the meat industry highlighted the period of 7–15 days after weaning (56.6% in meat flocks vs. 32.3% in dairy flocks), while a significantly higher percentage of veterinarians from the dairy industry indicated the period of 1–2 months after weaning (33.9% in dairy flocks vs. 6.6% in meat flocks) (*p* < 0.01) (Figures 2A,B). In the feedlots, although no statistical analysis could be performed, more than 40% of the veterinarians (46.2%, 6/13) agreed that 7–15 days after weaning is the more problematic period for coccidiosis, similar to veterinarians who worked with meat flocks.

**Figure 3:**
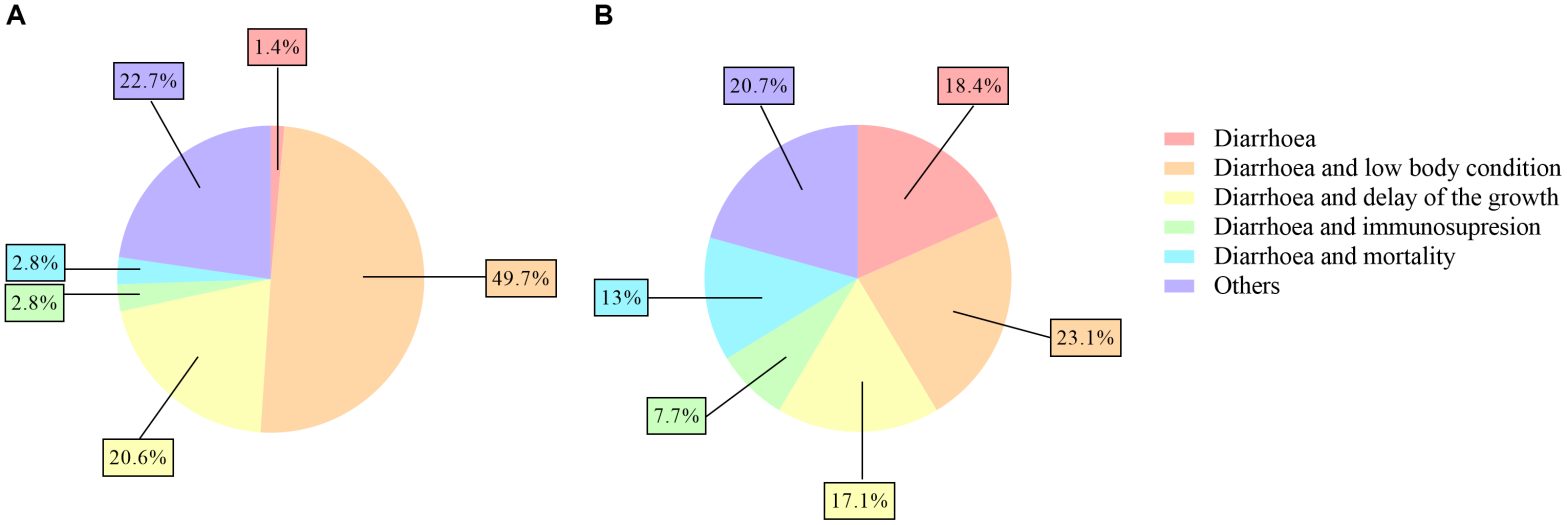
Pie charts of the signs associated with ovine coccidiosis reported by veterinarians **(A)** and farmers **(B)**.

Regarding clinical signs, a significantly higher percentage of veterinarians (49.6%, 70/141) answered that the main clinical signs of coccidiosis were diarrhea and poor body condition compared to farmers (23.1%, 39/169) (*p* < 0.05). In feedlots, 46.1% (6/13) of the veterinarians also recognized coccidiosis by diarrhea and poor body condition. However, a significantly higher percentage of farmers (39.1%, 66/169) answered that the main clinical sign was only diarrhea compared to veterinarians (7.1%, 10/141) (*p* < 0.0001). In addition, a significantly higher percentage of farmers (13%, 22/169) answered that the main clinical signs were diarrhea and mortality compared to veterinarians (2.8%, 4/141) (*p* < 0.01) (Figures 3A,B).

Regarding risk factors, most of the veterinarians (51.7%, 73/141) recognized that the flocks with a higher incidence of coccidiosis were those without cleaning and disinfection programs, followed by those that experienced overcrowding (22.7%, 32/141), those that included a large number of animals (22%, 31/141) and those with the presence of other diseases (3.6%, 5/141).

### 3.4 Diagnosis of *Eimeria* spp. infection

In meat and dairy flocks, most of the veterinarians (55.3%, 78/141) and farmers (62.7%, 106/169) recognized that they did not routinely perform coprological studies or did so sporadically (15.6%, 22/141, and 21.9%, 37/169, for veterinarians and farmers, respectively). A higher percentage of veterinarians carried out coprological studies once or twice a year (22%, 31/141) compared to farmers (9.5%, 16/169) (*p* < 0.01) (Table 4). Comparing production purposes, in feedlots, diagnoses seemed to be more common than in meat and dairy flocks, with 53.8% (7/13) of veterinarians diagnosing once or twice a year or in each lambing season/lot in the feedlots in comparison with 29.1% (41/141) in meat and dairy flocks.

**Table 4:** Diagnosis and control of ovine coccidiosis in meat and dairy flocks in Spain.

Category	Questions	Answers	Veterinarians	Farmers
Diagnosis	Do you perform coprological studies to monitor coccidiosis?	Not routinely	55.3% (78/141)	62.7% (106/169)
		Sporadically	15.6% (22/141)	21.9% (37/169)
		Once or twice a year	22% (31/141)	9.5% (16/169)
		In each lambing season/lot	7.1% (10/141)	5.9% (10/169)
	Do you order species identification?	No, never	68.9% (82/119)	ND
		Only in the initial diagnosis	21% (25/119)	ND
		Yes, routinely	10.1% (12/119)	ND
	At what oocyst count do you decide to treat for coccidial infection?	Up to 500 opg	31.9% (38/119)	ND
		501–1,000 opg	35.3% (42/119)	ND
		1,001–5,000 opg	22.7% (27/119)	ND
		>5,000 opg	10.1% (12/119)	ND
Control	Which have been your criteria for diagnosing and treating coccidiosis?	Compatible clinical signs without coprological studies	47.5% (67/141)	51.5% (87/169)
		Compatible clinical signs and presence of oocysts in feces	34.8% (49/141)	22.5% (38/169)
		Presence of oocysts in feces	5.7% (8/141)	6.5% (11/169)
		Systemically for all lots without coprological studies	12% (17/141)	19.5% (33/169)
	Which treatment/s do you use for coccidiosis?	Oral anticoccidials	46% (64/139)	43.8% (74/169)
		Medicated feed	15.1% (21/139)	14.8% (25/169)
		Combined treatment	18% (25/139)	21.3% (36/169)
		I do not usually treat, I only implement management measures	20.2% (28/139)	17.1% (29/169)
		I have not needed to apply any treatment	0.7% (1/139)	1.2% (2/169)
		Others	0% (0/139)	1.8% (3/169)
	When do you apply oral anticoccidials?	One week before weaning	35% (41/117)	20.4% (22/108)
		At the time of weaning	17.1% (20/117)	25.9% (28/108)
		15 days after weaning	10.3% (12/117)	8.3% (9/108)
		When compatible clinical signs appear	37.6% (44/117)	45.4% (49/108)
	How many doses of oral anticoccidials do you apply?	Single dose	67.5% (79/117)	67.3% (70/104)
		Two doses	22.2% (26/117)	23.1% (24/104)
		Some lots with more than two doses	10.3% (12/117)	9.6% (10/104)
	Who administers the oral anticoccidials?	Breeder	66.4% (77/116)	81.5% (84/103)
		Veterinarian	17.2% (20/116)	7.8% (8/103)
		Sometimes the veterinarian and sometimes the breeder	16.4% (19/116)	10.7% (11/103)
	How do you calculate the dose of oral anticoccidials?	I weigh several animals to establish an average weight	6% (7/116)	17.5% (18/103)
		I weigh the heaviest animal	14.7% (17/116)	2.9% (3/103)
		I estimate an average weight according to the age of the animals	25% (29/116)	29.1% (30/103)
		Visual estimation of the weight	54.3% (63/116)	50.5% (52/103)
	When do you start administering medicated feed?	One week before weaning	43.5% (20/46)	24.6% (15/61)
		At the time of weaning	21.7% (10/46)	52.5% (32/61)
		15 days after weaning	8.7% (4/46)	9.8% (6/61)
		When compatible clinical signs appear	26.1% (12/46)	13.1% (8/61)
	How long do you apply the medicated feed?	Less than 28 days	10.9% (5/46)	39% (23/59)
		Between 28 and 30 days	56.5% (26/46)	27.1% (16/59)
		Between 31 and 45 days	17.4% (8/46)	23.7% (14/59)
		Until the lot of feed runs out	15.2% (7/46)	10.2% (6/59)
	Which management measures do you implement?	Cleaning/disinfection	21.5% (29/135)	46.2% (73/158)
		Cleaning/disinfection and measures to minimize stress	48.1% (65/135)	15.2% (24/158)
		I do not implement management measures	5.9% (8/135)	21.5% (34/158)
		Others	24.5% (33/135)	17.1% (27/158)
	Which disinfectant do you use?	Quaternary ammoniums or quaternary ammoniums and others	25% (29/116)	23.6% (25/106)
		Peroxides or peroxides and others	54.3% (63/116)	47.2% (50/106)
		Peroxides and quaternary ammoniums	17.2% (20/116)	4.7% (5/106)
		Others	3.5% (4/116)	22.5% (26/116)

ND: question not included in the questionnaire.

Among veterinarians who performed coprological studies, 68.9% (82/119) did not order species identification, 10.1% (12/119) carried out species identification routinely, and 21% (25/119) ordered species identification only in the initial diagnosis. Among those veterinarians who performed coprological studies, 32% (38/119), 35.3% (42/119), 22.7% (27/119), and 10% (12/119) implemented treatment with a number of *Eimeria* spp. oocysts per gram of feces (opg) of ≤500 opg, ≥501–≤1,000 opg, ≥1,001–≤5,000 opg, and ≥5,001 opg, respectively (Table 4).

### 3.5 Control measures for ovine coccidiosis

**Figure 4:**
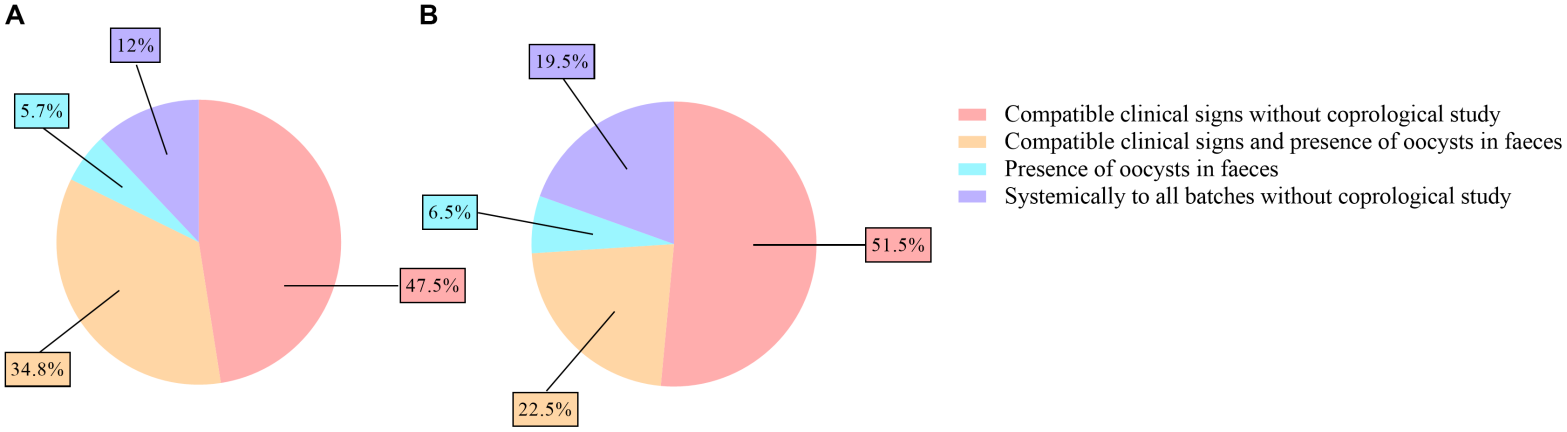
Pie charts of the criteria for the treatment of ovine coccidiosis reported by veterinarians **(A)** and farmers **(B)**.

One of the approaches for the control of ovine coccidiosis consists of the use of treatments, which were applied based on compatible clinical signs by 82.3% (116/141) and 74% (125/169) of the veterinarians and farmers, respectively, even without evidencing the presence of oocysts through a coprological study by 47.5% (67/141) and 51.5% (87/169) of them. In contrast, 5.7% (8/141) and 6.5% (11/169) of the veterinarians and farmers, respectively, determined the use of treatments based only on the detection of oocysts in the feces. Finally, the application of treatment to all lots without a coprological study was conducted by 12% (17/141) of the veterinarians and 19.5% (33/169) of the farmers (Figures 4A,B and Table 4). No significant differences were found in the criteria for treatment applied by veterinarians and farmers. However, the criteria for treatment were influenced by the flock size. Thus, treatment based on compatible clinical signs without coprological studies were more frequent in flocks with less than 500 animals (68.5%, 37/54) or with 500–1,000 animals (54%, 34/63) compared to flocks with more than 1,500 animals (17.2%, 5/29) (*p* < 0.01), where most of the farmers treated based on compatible clinical signs and the presence of oocysts in feces (41.4%, 12/29) or systematically to all lots without coprological studies (31%, 9/29).

Treatment was used by 79.1% (110/139) of the veterinarians and 79.9% (135/169) of the farmers. There were no significant differences in application or in the kind of treatment between veterinarians and farmers (Table 4). However, similar to the criteria for treatment, there were significant differences in whether treatment was used when comparing the size of the flocks. Thus, a higher percentage (31.5%, 17/54) of the flocks with less than 500 animals did not usually receive treatment compared to flocks with more than 1,000 animals (7.7%, 4/52) (*p* < 0.05).

Oral anticoccidials (diclazuril and toltrazuril) were the preferred anticoccidials, used by 46% (64/139) of the veterinarians and 43.8% (74/169) of the farmers. They were mainly used when compatible clinical signs appeared (37.6%, 44/117, of veterinarians and 45.4%, 49/108, of farmers), followed by 1 week before weaning for veterinarians (35%, 41/117) and at the time of weaning for farmers (25.9%, 28/108), although no significant differences were found between them (Table 4). Separating by production purpose, in meat flocks, oral anticoccidials were administered more frequently when compatible clinical signs appeared (44.4%, 28/63) than in dairy flocks (29.6%, 16/54) (*p* < 0.01). The number of doses did not differ between veterinarians and farmers, with most of them administering a single dose (67.5%, 79/117, of veterinarians and 67.3%, 70/104, of farmers) (Table 4). Two doses were applied more frequently by veterinarians working with dairy flocks (65.4%, 17/26) or on feedlots (60%, 6/10) than by veterinarians working with meat flocks (34.6%, 9/26) (*p* < 0.05). Oral anticoccidials were applied in 66–81% of the cases by farmers (Table 4). However, a significantly higher percentage of veterinarians applied oral anticoccidials in meat flocks (15.9%, 7/44) compared to dairy flocks (1.7%, 1/59) (*p* < 0.01). When administering them, the most common way to calculate the dose was to visually estimate the weight of the animals (54.3%, 63/116, of veterinarians and 50.5%, 52/103, of farmers) (Table 4). In addition, visual estimation was more common among farmers who treated animals without coprological studies (55.5%, 40/72) than among those who performed coprological studies (38.7%, 12/31) (*p* < 0.05).

Regarding medicated feed (decoquinate), the main period of application was at the time of weaning or 1 week before weaning (65.2%, 30/46, of veterinarians and 77.1%, 47/61, of farmers); however, a relatively substantial percentage administered medicated feed when compatible clinical signs appeared (26.1%, 12/46, of the veterinarians and 13.1%, 8/61, of the farmers) (Table 4). Regarding the duration of treatment with medicated feed, veterinarians administered this treatment more frequently for 28–30 days (56.5%, 26/46) than farmers (27.1%, 16/59), while farmers more frequently administered it for less than 28 days (39%, 23/59) than veterinarians (10.9%, 5/46) (*p* < 0.001) (Table 4). Moreover, a higher proportion of farmers with meat flocks administered it for less than 28 days (70%, 14/20) compared to farmers with dairy flocks (23.1%, 9/39) (*p* < 0.05). Finally, compared with the percentage of replacement, flocks with less than 25% replacement (smallest flocks) more frequently received medicated feed for less than 28 days (59.4%, 19/32) compared to flocks with more than 25% replacement (largest flocks) (14.8%, 4/27) (*p* < 0.01).

Another approach for the control of coccidiosis is cleaning/disinfection and management measures. A significantly greater proportion of veterinarians (48.1%, 65/135) than farmers (15.2%, 24/158) (*p* < 0.0001) answered that they controlled coccidiosis by combining cleaning and disinfection of the paddocks and management measures. On the other hand, control measures were more commonly implemented only by cleaning and disinfection of the paddocks by farmers (46.2%, 73/158) than by veterinarians (21.5%, 29/135) (*p* < 0.0001) (Table 4). Comparing production systems, a greater proportion of farmers with dairy flocks (21.6%, 21/97) combined cleaning and disinfection of the paddocks and management measures than farmers with meat flocks (4.9%, 3/61) (*p* < 0.01). Regarding disinfection, peroxides were the most commonly used disinfectants (54.3%, 63/116, of veterinarians and 47.2%, 50/106, of farmers), with the combination of peroxides and quaternary ammoniums more frequently applied by veterinarians (17.2%, 20/116) (*p* < 0.05). Farmers more often chose “other disinfectants” (24.5%, 26/106) (*p* < 0.0001).

## 4 Discussion

Coccidiosis affects ovine flocks worldwide (7), including some major sheep-producing countries in the Mediterranean area, such as Spain, causing mortality and low growth rates among lambs (25). However, the knowledge concerning infection dynamics and the extent to which the tools for the diagnosis and control of the disease are put into practice by veterinarians and farmers is not known thus far. Spain is the country with the greatest ovine census in the European Union (26), and breeding traditionally has two different purposes, meat or dairy production. In addition, there are feedlots that concentrate a large number of lambs for fattening (25); however, there are few feedlots in Spain, and therefore, the questionnaires from feedlots were not included in the statistical analysis in the present study. The breeding flocks included in the questionnaires were representative of those in Spain, as according to official published data (27), the number of animals in each flock, for both breeding purposes, matches the data presented here. In addition, dairy flocks have a high level of intensification and stocking rates, which have been identified as risk factors for coccidiosis due to facilitated transmission (13). Regarding the replacement rate, in breeding flocks, on average, 15 and 25% of the sheep would be replaced annually in meat and dairy flocks, respectively (28, 29); therefore, the purpose of breeding the sheep determines the productive lifespan of sheep and consequently the replacement rate. Similarly, in this study, an association between the breeding purpose and the replacement rate was found.

Focusing on the main diseases in the flocks, similar to previous studies (25, 30), ORC was also highlighted as an important disease. In addition, according to our results, ORC seemed to be more problematic in meat flocks and feedlots since in these, the lambs are housed beyond 2 months of age, a period in which ORC has a higher morbidity (30). Another infectious disease marked as relevant by questionnaire participants was neonatal diarrhea, which has a high incidence in Spain (31). In addition, according to the results of the questionnaires, neonatal diarrhea seemed to be more frequent in dairy flocks since lambs are sold around 1 month of age (mainly to feedlots), which precludes the occurrence of other diseases that usually appear at older ages (such as ORC and coccidiosis). Finally, similar to other surveys in Norway in which 54% of the farmers reported ovine coccidiosis as relevant (24), in this study, coccidiosis was reported by 33–34% of the respondents. Apart from the direct clinical and economic consequences of *Eimeria* spp. infection, coccidiosis leads to immunosuppression in lambs, and consequently, lambs with coccidiosis have a 1.84-fold higher risk of suffering ORC (25).

Analyzing the risk period for ovine coccidiosis, lambs are usually infected with *Eimeria* spp. within the first days of life, starting oocyst excretion at approximately 2–3 weeks of life; however, the dynamics of oocyst excretion and the clinical/subclinical presentation depend on the management system (6). With the stress associated with weaning and regrouping, oocyst excretion peaks (1), leading to the first clinical cases (25, 32), although the maximum incidence of coccidiosis is reached after weaning (25, 33). In intensive dairy flocks in Spain, lambs are housed together and are fed artificial milk just after colostrum intake, usually following recommended hygienic measures until weaning, which could lead to low oocyst contamination, leading to a delay in the occurrence of coccidiosis until 1–2 months after weaning. In contrast, in meat flocks in Spain, lambs are suckled by the dams until weaning, usually under unfavorable hygienic conditions, which could lead to an increase in the likelihood of contact with oocysts before weaning and consequently the period of greatest risk of coccidiosis just after weaning (7–15 days after weaning), as was stated in the questionnaires in the present study.

The main clinical sign of ovine coccidiosis is diarrhea (32); however, as was observed in the present study from the veterinarians’ questionnaires, subclinical coccidiosis (whose main signs are delay of growth and poor body condition) may lead to higher production losses than clinical coccidiosis (2, 7). Cleaning and disinfection of the flocks have been largely studied as protective factors against ovine coccidiosis (1, 34), which is the reason why more than 50% of the veterinarians highlighted them in this study, emphasizing that they are of the utmost importance to interrupt orofecal transmission of *Eimeria* spp. A high animal density is a well-known risk factor for coccidiosis, contributing to increased infection pressure (13, 14, 35, 36), and the results of this study also support this finding. Space constraints may be more common in dairy flocks (intensive management) in Spain; however, in meat flocks (extensive management) in Spain with reduced pasture availability, coccidiosis can also be prevalent (37). Finally, in the largest flocks, a higher incidence of clinical and subclinical coccidiosis has been described (14, 24), and in this study, 22% of veterinarians stated that they had observed a higher incidence of coccidiosis in the flocks with the greatest number of animals.

Regulation (EU) 2019/6 aimed to avoid the prophylactic use of antimicrobials for which diagnostics are essential. The diagnosis of ovine coccidiosis based on the observation of oocysts in feces has been described as rare after interviewing Norwegian farmers, with only 12% of them submitting fecal samples to diagnostic laboratories (24). Similarly, the present study also revealed a low number of diagnoses, with 70–84% of veterinarians/farmers not conducting diagnoses routinely or doing so sporadically, although in feedlots, the frequency of diagnoses seemed to be slightly higher. In the present study, the reason for diagnosis was not tracked; however, in (24), most of the diagnoses were for surveillance (65.4%), with 18.4% due to disease and 16.2% a combination of these. Along with the detection of oocysts, species identification using morphometric variables (preceded by sporulation of the oocysts) is essential for the following reasons: (i) *Eimeria* spp. infections are present in most flocks (38), and a 100% individual prevalence and a 100% concomitant infections with more than one *Eimeria* species have been described in some flocks (14), (ii) *Eimeria* spp. show different pathogenicity (9, 10), and replication potential (1), but 68% of the veterinarians in the present study did not order species identification. In addition, species identification should always be requested since the prevalence of different *Eimeria* spp. differs with the age of the animals (4) or in different lambing seasons (6); however, 21% of the veterinarians from this study ordered species identification only in the initial diagnosis. The interpretation of oocyst counts is difficult, and the number of opg does not necessarily correlate with the clinical outcome (6), since a relatively large number of oocysts from low pathogenicity species could be detected in apparently healthy animals (7, 39, 40), and mixed infections increase oocyst excretion (41). In this study, we found a wide diversity of opg in establishing treatment, which may be due to the lack of an exact number of opg for relevant excretion (7), although some authors established counts higher than 500 opg as an indication of active infection or counts higher than 5,000 opg as an indication of clinical disease (40, 42).

For the control of the infection, the use of drugs was based in a very high proportion on the presence of clinical signs, contrasting with previous studies in which the use of therapeutics was rare and they were applied commonly as metaphylactic treatment (24). Therapeutic treatment is unsuccessful since intestinal damage and the excretion of oocysts have already taken place, and the only benefit would be a lower excretion of oocysts (7, 34). Treatment without a previous diagnosis was also very common in this study and may have led to unnecessary treatment, even increasing the likelihood of antimicrobial resistance; only those flocks that were diagnosed were able to receive precise treatment, including subclinical cases. The proportion of flocks that received treatment (79%) was very similar to that described by (24), although this proportion was lower for smaller flocks as also described (24). Oral anticoccidials (diclazuril and toltrazuril) were more commonly used than medicated feed (decoquinate); however, the timing of application was poor in most cases (when compatible clinical signs appeared), contrasting with that described by (24) and probably increasing the risk of treatment failure (43). Interestingly, meat flocks were more likely than dairy flocks to receive oral anticoccidials in clinical cases, which may be explained by a greater incidence of subclinical coccidiosis in larger flocks (which are usually dairy flocks) (14, 24). A single administration of oral anticoccidials around the time of weaning is usually enough to reduce oocyst excretion and to improve growth parameters (3), which is the reason why in this study and in (24), most of the flocks received a single dose. However, some studies described better outcomes in animals treated twice (35, 44), which is more common in environments that are heavily contaminated with oocysts such as dairy flocks and feedlots. Following good practices, weighing individual animals, or at least several of them, is the most recommended method to calculate the dose of antimicrobial agents; however, from a practical point of view, it entails strict management, and very few flocks practice this. Visual estimation of the weight is the most common practice and is even more frequently practiced in smaller flocks, similar to previous studies (24), and may lead to incorrect dosing (increasing the likelihood of antimicrobial resistance). Other methods, such as weighing the heaviest lamb, are occasionally used and could lead to overdosing, making the drug more efficacious in the primoinfection and generating lower immune responses to protect against subsequent infections (18). For medicated feed (decoquinate), a proportion of the treatments were applied when clinical signs appeared, although less frequently than for oral anticoccidials. Medicated feed must be maintained continuously for at least 28 days since decoquinate does not kill *Eimeria* spp. but inhibits their development (7, 16, 45); however, in meat flocks and small flocks, it is usually administered for a shorter time, compromising its efficacy.

Management measures to reduce animal stress (weaning, regrouping of animals and animal density) seems to be more commonly implemented in dairy flocks, probably because of their larger size and stocking rates, and helps to minimize the use of drugs, with a combination with cleaning/disinfection being a better approach. In addition, inadequate cleaning can reduce the efficacy of disinfectants, but the use of boiling water along with quaternary ammoniums and peroxides is a suitable protocol for the inactivation of oocysts (46), which is well known to veterinarians and almost unknown to farmers.

Collecting data is an important step in the identification of weaknesses, and the questionnaires from the present study clarified the specific issues to be addressed to improve the control of ovine coccidiosis. The control of ovine coccidiosis was severely hampered by a lack of diagnoses, and although treatments were largely applied, in many flocks, inappropriate clinical criteria for treatment were commonly used. Differences between meat and dairy flocks were identified, concluding that in dairy flocks, there was a better implementation of management measures (cleaning and management measures to reduce stress in the animals), which could be the reason for the emergence of coccidiosis long after weaning. In contrast, in meat flocks, the use of treatment was less extensive, although it was more commonly applied following improper methods such as the use of anticoccidials for clinical cases and of coccidiostats during a short period of time. As required by Regulation 2019/6, the treatments must not be used prophylactically, and for *Eimeria* spp. infections, treatment should be applied during the prepatent period (metaphylactic). Therefore, diagnoses of *Eimeria* spp. infections in each individual flock as well as field studies in a representative number of meat and dairy ovine flocks in Spain are needed to provide information on the dynamics of *Eimeria* spp. infections to establish the best timing for intervention.

## Data availability statement

The original contributions presented in the study are included in the article/Supplementary Material, further inquiries can be directed to the corresponding authors.

## Ethics statement

Medical research was not carried out; therefore, ethics approval was not needed. Anonymity was maintained in the study; therefore, written informed consent was not needed.

## Author contributions

RS-S: Conceptualization, Formal analysis, Investigation, Methodology, Writing – original draft. JG: Conceptualization, Data curation, Funding acquisition, Investigation, Methodology, Resources, Writing – review & editing. JB-C: Conceptualization, Data curation, Funding acquisition, Investigation, Methodology, Writing – review & editing. MM-S: Conceptualization, Data curation, Methodology, Resources, Writing – review & editing. SC-A: Writing – review & editing, Formal analysis. LE: Conceptualization, Funding acquisition, Investigation, Writing – review & editing, Data curation, Methodology. IF: Conceptualization, Funding acquisition, Investigation, Project administration, Resources, Supervision, Writing – review & editing. LO-M: Conceptualization, Funding acquisition, Investigation, Project administration, Resources, Supervision, Writing – review & editing.

## Footnotes

^1^https://www.getfeedback.com/en/
