# Supplementary material for: A questionnaire-based survey in Spain provides relevant information to improve the control of ovine coccidiosis
Source: Front Vet Sci. 2023 Dec 6;10:1326431. doi: 10.3389/fvets.2023.1326431 (PMC10730930; doi:10.3389/fvets.2023.1326431)
Supplement: Supplementary file 1 [file Table_1.DOCX]

**Supplementary file 1. Combination of answers in multi-response questions.**

| **Questions** | **Combined answers** |
| --- | --- |
| 1. Select the two diseases affecting sheep flocks you are most concerned about | 1. a) Neonatal diarrhoea and ovine respiratory complex b) Neonatal diarrhoea and coccidiosis or neonatal diarrhoea and abortion 2. c) Ovine respiratory complex or ovine respiratory complex and coccidiosis or ovine respiratory complex and abortion 3. d) Others |
| 1. What are the two main signs by which you suspect coccidiosis? | a) Diarrhoea b) Diarrhoea and low body condition c) Diarrhoea and delay of growth d) Diarrhoea and immunosupresion e) Diarrhoea and mortality f) Others |
| 1. In which kind of flocks do you have more coccidiosis? | a) Flocks with absence of cleaning and disinfection programs for paddocks or flocks with absence of cleaning and disinfection programs for paddocks and others b) Flocks with presence of other diseases or others c) Flocks with the highest census of animals or flocks with the highest census of animals and others  d) In lots with problems of overcrowding and heterogeneity or in lotes with problems of overcrowding and heterogeneity and others |
| 21) Which management measures do you apply? | a) Cleaning and disinfection of the paddocks b) Cleaning and disinfection of the paddocks and measures to minimize stress (animal density, weaning, etc) c) I do not apply management measures d) Others |
| 22) Which disinfectant do you use? | 1. a) Quaternary ammoniums or quaternary ammoniums and others b) Peroxides or peroxides and others c) Peroxides and quaternary ammoniums d) Others |
