# Supplementary material for: A questionnaire-based survey in Spain provides relevant information to improve the control of ovine coccidiosis
Source: Front Vet Sci. 2023 Dec 6;10:1326431. doi: 10.3389/fvets.2023.1326431 (PMC10730930; doi:10.3389/fvets.2023.1326431)
Supplement: Supplementary file 2 [file Table_2.DOCX]

**Supplementary file 2. P values of the comparisons of the questions asked to veterinarians**

| **Question^a^** | **2** | **6** | **7** | **8** | **9** | **10** | **11** | **12** | **13** | **14** | **15** | **16** | **17** | **18** | **19** | **20** | **21** | **22** |
| --- | --- | --- | --- | --- | --- | --- | --- | --- | --- | --- | --- | --- | --- | --- | --- | --- | --- | --- |
| **2** |  | | |  |  |  |  |  |  |  |  |  |  |  |  |  |  |  |
| **6** | 0.016* |  |  |  |  |  |  |  |  |  |  |  |  |  |  |  |  |  |
| **7** | 0.095 | 0.299 |  |  |  |  |  |  |  |  |  |  |  |  |  |  |  |  |
| **8** | 0.894 | 0.909 | 0.739 |  |  |  |  |  |  |  |  |  |  |  |  |  |  |  |
| **9** | <0.001* | 0.432 | 0.012* | 0.809 |  |  |  |  |  |  |  |  |  |  |  |  |  |  |
| **10** | 0.187 | 0.619 | 0.900 | 0.141 | 0.141 |  |  |  |  |  |  |  |  |  |  |  |  |  |
| **11** | 0.045* | 0.983 | 0.547 | 0.236 | <0.001* | <0.001* |  |  |  |  |  |  |  |  |  |  |  |  |
| **12** | 0.363 | 0.374 | 0.995 | 0.080 | 0.617 | 0.056 | 0.017* |  |  |  |  |  |  |  |  |  |  |  |
| **13** | 0.892 | 0.821 | 0.591 | 0.393 | 0.361 | <0.001* | 0.057 | 0.200 |  |  |  |  |  |  |  |  |  |  |
| **14** | 0.984 | 0.352 | 0.971 | 0.077 | 0.757 | 0.636 | 0.432 | 0.902 | 0.847 |  |  |  |  |  |  |  |  |  |
| **15** | 0.013* | 0.222 | 0.251 | 0.570 | <0.001* | 0.071 | 0.142 | 0.618 | 0.066 | 0.126 |  |  |  |  |  |  |  |  |
| **16** | 0.013* | 0.432 | 0.122 | 0.908 | 0.812 | 0.148 | 0.946 | 0.332 | 0.234 | 0.346 | 0.331 |  |  |  |  |  |  |  |
| **17** | 0.642 | 0.961 | 0.205 | 0.652 | 0.146 | 0.020* | 0.925 | 0.588 | 0.654 | 0.769 | 0.563 | 0.920 |  |  |  |  |  |  |
| **18** | 0.193 | 0.356 | 0.767 | 0.373 | 0.629 | 0.406 | 0.417 | 0.154 | 0.085 | 0.052 | 0.087 | 0.524 | 0.332 |  |  |  |  |  |
| **19** | 0.384 | 0.646 | 0.353 | 0.729 | 0.301 | 0.130 | 0.195 | 0.085 | 0.288 | 0.332 | 0.603 | 0.501 | 0.597 | 0.266 |  |  |  |  |
| **20** | 0.823 | 0.046* | 0.963 | 0.644 | 0.159 | 0.088 | 0.713 | 0.042* | 0.869 | 0.125 | 0.913 | 0.566 | 0.768 | 0.708 | 0.051 |  |  |  |
| **21** | 0.814 | 0.044* | 0.964 | 0.903 | 0.259 | 0.466 | 0.279 | 0.719 | 0.135 | 0.066 | 0.166 | 0.551 | 0.098 | 0.091 | 0.685 | 0.203 |  |  |
| **22** | 0.828 | 0.601 | 0.318 | 0.483 | 0.439 | 0.369 | 0.490 | 0.141 | 0.276 | 0.079 | 0.182 | 0.966 | 0.248 | 0.147 | 0.027* | 0.338 | 0.226 |  |

^a^ The questions and answers are listed in Table 1.

* P < 0.05
