# Supplementary material for: A questionnaire-based survey in Spain provides relevant information to improve the control of ovine coccidiosis
Source: Front Vet Sci. 2023 Dec 6;10:1326431. doi: 10.3389/fvets.2023.1326431 (PMC10730930; doi:10.3389/fvets.2023.1326431)
Supplement: Supplementary file 3 [file Table_3.DOCX]

**Supplementary file 3. P values of the comparisons of the questions asked to farmers**

| **Question^a^** | **2** | **3** | **4** | **5** | **6** | **7** | **9** | **10** | **13** | **14** | **15** | **16** | **17** | **18** | **19** | **20** | **21** | **22** |
| --- | --- | --- | --- | --- | --- | --- | --- | --- | --- | --- | --- | --- | --- | --- | --- | --- | --- | --- |
| **2** |  | |  |  |  |  |  |  |  |  |  |  |  |  |  |  |  |  |
| **3** | 0.048* |  |  |  |  |  |  |  |  |  |  |  |  |  |  |  |  |  |
| **4** | <0.001* | 0.174 |  |  |  |  |  |  |  |  |  |  |  |  |  |  |  |  |
| **5** | <0.001* | <0.001* | <0.001* |  |  |  |  |  |  |  |  |  |  |  |  |  |  |  |
| **6** | 0.089 | 0.562 | 0.195 | 0.896 |  |  | | | | | | | | | | | | |
| **7** | 0.748 | 0.674 | 0.907 | 0.057 | 0.679 |  |  |  |  |  |  |  |  |  |  |  |  |  |
| **9** | 0.008* | 0.812 | 0.292 | 0.259 | 0.393 | 0.226 |  |  |  |  |  |  |  |  |  |  |  |  |
| **10** | 0.111 | 0.274 | 0.004* | 0.109 | 0.119 | 0.946 | 0.305 |  |  |  |  |  |  |  |  |  |  |  |
| **13** | 0.069 | 0.007* | 0.169 | 0.012* | 0.582 | 0.362 | 0.314 | <0.001* |  |  |  |  |  |  |  |  |  |  |
| **14** | 0.100 | 0.007* | 0.575 | 0.042* | 0.529 | 0.259 | 0.378 | 0.180 | 0.314 |  |  | | | | | | | |
| **15** | 0.105 | 0.138 | 0.789 | 0.131 | 0.824 | 0.492 | 0.012* | 0.011* | 0.870 | 0.286 |  |  |  |  |  |  |  |  |
| **16** | 0.289 | 0.213 | 0.130 | 0.233 | 0.448 | 0.029* | 0.626 | 0.916 | 0.638 | 0.703 | 0.193 |  |  |  |  |  |  |  |
| **17** | 0.015* | 0.395 | 0.543 | 0.701 | 0.667 | 0.328 | 0.306 | 0.056 | 0.046* | 0.230 | 0.306 | 0.883 |  |  |  |  |  |  |
| **18** | 0.165 | 0.771 | 0.398 | 0.319 | 0.869 | 0.078 | 0.180 | 0.018* | 0.004* | 0.348 | 0.631 | 0.334 | 0.705 |  |  |  |  |  |
| **19** | 0.491 | 0.178 | 0.505 | 0.446 | 0.507 | 0.666 | 0.074 | 0.144 | 0.847 | 0.364 | 0.115 | 0.123 | 0.718 | 0.732 |  |  |  |  |
| **20** | <0.001* | 0.946 | 0.008* | 0.394 | 0.970 | 0.104 | 0.076 | 0.432 | 0.295 | 0.044* | 0.410 | 0.876 | 0.825 | 0.083 | 0.057 |  |  |  |
| **21** | 0.038* | 0.357 | 0.179 | 0.453 | 0.027* | 0.654 | 0.733 | 0.014* | 0.758 | 0.362 | 0.937 | 0.779 | 0.814 | 0.122 | 0.310 | 0.454 |  |  |
| **22** | 0.152 | 0.186 | 0.313 | 0.364 | 0.906 | 0.320 | 0.315 | 0.264 | 0.001* | 0.247 | 0.037* | 0.452 | 0.161 | 0.125 | 0.600 | 0.508 | 0.311 |  |

^a^ The questions and answers are listed in Table 1.

* P < 0.05
