# Supplementary material for: A questionnaire-based survey in Spain provides relevant information to improve the control of ovine coccidiosis
Source: Front Vet Sci. 2023 Dec 6;10:1326431. doi: 10.3389/fvets.2023.1326431 (PMC10730930; doi:10.3389/fvets.2023.1326431)
Supplement: Supplementary file 4 [file Table_4.DOCX]

**Supplementary file 4. P values of the comparisons of the questions asked to veterinarians and farmers**

| **Question^a^** | **2** | **6** | **7** | **9** | **10** | **13** | **14** | **15** | **16** | **17** | **18** | **19** | **20** | **21** | **22** |
| --- | --- | --- | --- | --- | --- | --- | --- | --- | --- | --- | --- | --- | --- | --- | --- |
| **2** | 0.012* |  |  |  |  |  |  |  |  |  |  |  |  |  |  |
| **6** |  | <0.001* |  |  |  |  |  |  |  |  |  |  |  |  |  |
| **7** |  | | <0.001* |  |  |  |  |  |  |  |  |  |  |  |  |
| **9** |  |  |  | 0.002* |  |  |  |  |  |  |  |  |  |  |  |
| **10** |  |  |  |  | 0.015* |  |  |  |  |  |  |  |  |  |  |
| **13** |  |  |  |  |  | 0.068 |  |  |  |  |  |  |  |  |  |
| **14** |  |  |  |  |  |  | 0.620 |  |  |  |  |  |  |  |  |
| **15** |  |  |  |  |  |  |  | 0.098 |  |  |  |  |  |  |  |
| **16** |  |  |  |  |  |  |  |  | 0.980 |  |  |  |  |  |  |
| **17** |  |  |  |  |  |  |  |  |  | 0.032* |  |  |  |  |  |
| **18** |  |  |  |  |  |  |  |  |  |  | 0.002* |  |  |  |  |
| **19** |  |  |  |  |  |  |  |  |  |  |  | 0.009* |  |  |  |
| **20** |  |  |  |  |  |  |  |  |  |  |  |  | 0.003* |  |  |
| **21** |  |  |  |  |  |  |  |  |  |  |  |  |  | <0.001* |  |
| **22** |  |  |  |  |  |  |  |  |  |  |  |  |  |  | <0.001* |

^a^ The questions and answers are listed in Table 1.

* P < 0.05
